# DerivaPredict: A User-Friendly Tool for Predicting and Evaluating Active Derivatives of Natural Products

**DOI:** 10.3390/molecules30081683

**Published:** 2025-04-09

**Authors:** Yu Song, Meng Zhang, Sihao Chang, Ganghui Chu, Hongchao Ji

**Affiliations:** 1 Laboratory of Xinjiang Native Medicinal and Edible Plant Resource Chemistry, College of Chemistry and Environmental Science, Kashi University, Kashi 844006, China; syusalutezdys@gmail.com; 2 Shenzhen Branch, Guangdong Laboratory for Lingnan Modern Agriculture, Genome Analysis Laboratory of the Ministry of Agriculture and Rural Affairs, Agricultural Genomics Institute at Shenzhen, Chinese Academy of Agricultural Sciences, Shenzhen 518120, Chinachangsihao@foxmail.com (S.C.)

**Keywords:** natural product derivatives, in silico molecular design, software engineering

## Abstract

While natural products and derivatives have been crucial in drug discovery, the current databases are limited to known compounds. There is a need for tools that can automatically generate and assess novel derivatives of natural products to enhance early-stage drug discovery. We present DerivaPredict (v1.0), a user-friendly tool that generates novel natural product derivatives through chemical and metabolic transformations. It predicts binding affinities using pretrained deep learning models and assesses drug-likeness via ADMET profiling. DerivaPredict is freely accessible with a source code on GitHub.

## 1. Introduction

Natural products have long been a cornerstone of drug discovery and development, offering an unparalleled diversity of chemical structures and biological activities. From traditional herbal remedies to modern pharmaceuticals, natural products have played a critical role in addressing human health challenges [1]. Iconic examples include paclitaxel (Taxol), a widely used chemotherapy agent derived from the Pacific yew tree, and digoxin, a cardiac glycoside sourced from the foxglove plant. These compounds underscore the immense therapeutic potential of natural products in treating diseases. Over the years, advancements in extraction techniques, synthetic chemistry, and biotechnological methods have expanded the scope of natural products research, allowing scientists to modify existing compounds and create derivatives with improved efficacy, safety, and pharmacokinetic profiles [2,3]. Structural modifications based on natural product scaffolds can lead to novel bioactive compounds, offering a rational approach to expanding chemical space and identifying new drug candidates. However, despite these technological advancements, the process of discovering and optimizing new drug candidates remains a formidable challenge.

Databases of natural products and their derivatives, such as SuperNatural [4], NPAtlas [5], and COCONUT [6], have provided the research community with molecular structures for drug screening. They are constructed by aggregating data from various sources, including experimental studies, literature reviews, and cheminformatics pipelines. Despite their comprehensive construction and utility, these databases are inherently limited to already-cataloged molecules. They reflect the extent of current knowledge and do not address the need for generating novel chemical entities.

Recently, computer-aided drug design has become a crucial component of modern drug discovery efforts, particularly in the development of bioactive compounds [7,8]. Notably, de novo drug design, including molecular generation, has gained significant attention for its ability to accelerate the discovery and optimization of novel ligands with desirable therapeutic properties [9,10]. Various machine learning frameworks and models have exhibited promising and efficient performances in drug-like molecule generation [11]. For example, sequence-based recurrent neural networks (RNNs) utilize sequential learning to generate molecular structures in the form of SMILES representations by grasping the patterns from existing molecules [12,13,14,15,16]. Variational autoencoders (VAEs) encode molecules into a continuous latent space, facilitating the smooth interpolation and modification of chemical structures [17,18,19,20,21]. Generative adversarial networks (GANs) employ a generator–discriminator framework to produce novel molecular structures that resemble real compounds [22,23,24,25,26]. Large language models (LLMs) utilize natural language processing capabilities to design and generate molecular structures based on textual prompts [27,28,29]. While these generative approaches have proven to be powerful tools for creating novel molecular structures, they are not specifically designed to produce derivatives of natural products. Furthermore, they typically operate without an explicit consideration of the biochemical transformation rules and reaction mechanisms that underlie the natural derivation of compounds, limiting their applicability in generating bioactive derivatives with realistic modifications.

To address this limitation, we introduce DerivaPredict, a computational framework specifically designed to facilitate the prediction and evaluation of natural product derivatives. Unlike generative models that create molecules without constraints, DerivaPredict incorporates curated chemical and biological reaction rules, ensuring that the generated derivatives align with the known reaction templates of biosynthetic and enzymatic transformations. It is important to note that the purpose of this work is not to predict natural product derivatives that may exist in the real world but rather to provide potential chemical structures for drug screening, which aligns with the goal of molecular generation algorithms. The key distinction is that it aims to expand the chemical space based on the existing natural product scaffolds and known reaction rules, which is beneficial for generating molecules with a lower synthetic complexity. Therefore, it can be used as a complementary solution for molecular generation tools for drug design.

Beyond the structure generation, DerivaPredict integrates state-of-the-art computational pipelines to evaluate the drug potential of the generated derivatives. It employs state-of-the-art machine learning models to predict binding affinities, providing insights into the likelihood of interaction with specific biological targets. Additionally, it conducts ADMET (absorption, distribution, metabolism, excretion, and toxicity) profiling to assess the pharmacokinetic and safety profiles of the compounds. It is important to emphasize that the primary purpose of DerivaPredict is to generate candidate derivative structures for drug screening. While the predicted results offer preliminary insights into the potential efficacy and safety of the compounds, they do not guarantee performance in in vivo activity experiments. Additional proteomic and cellular experiments are necessary [30,31,32].

## 2. Results

### 2.1. Software Integration

DerivaPredict is an open-source, user-friendly software tool designed to streamline natural product-based drug discovery. It features a graphical user interface (GUI) built using the QT framework. The software is designed to cater to both researchers with minimal computational expertise and advanced users who require flexibility and customizability.

**Figure 1:**
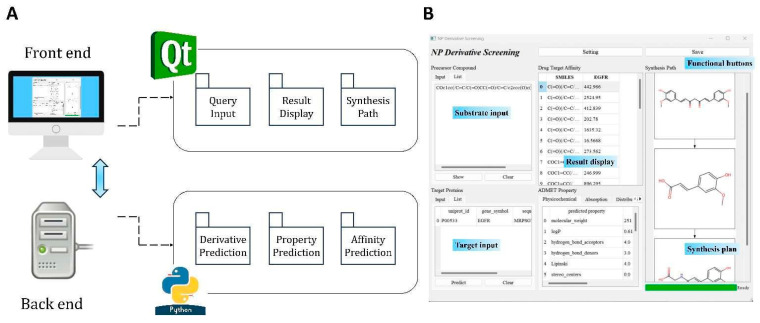
Overview of software integration. (**A**) Software architecture with front-end separation. (**B**) Graphical user interface screenshot of DerivaPredict software.

The software architecture, as illustrated in Figure 1, adopts a modular design where the front end and back end are fully independent. This separation ensures that updates or modifications to one component do not disrupt the other, enhancing the system’s reliability and maintainability. The front end provides a clean, interactive interface for users to input data, configure settings, and view results, while the back end performs the computational heavy lifting, including reaction rule applications, derivative generation, and molecular property predictions.

The modular structure of DerivaPredict also facilitates scalability and extensibility. Developers can easily integrate additional databases by replacing existing resource files without the need for extensive reengineering. This flexibility makes DerivaPredict a platform for ongoing development and collaboration, encouraging contributions from the research community to enhance its functionality further.

### 2.2. Software Functionalities

**Figure 2:**
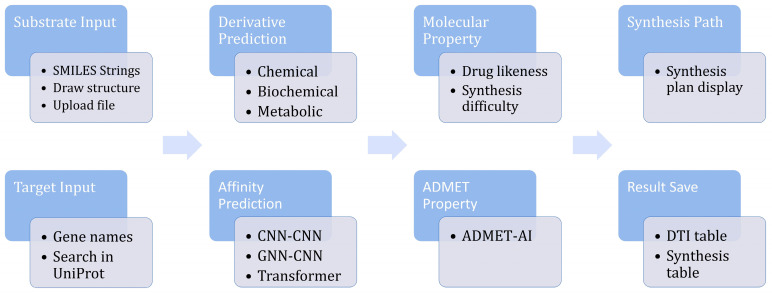
Workflow and functions of DerivaPredict software.

The GUI of DerivaPredict is shown in Figure 2. Researchers can start by inputting a natural product structure as the initial substrate. This can be accomplished in several ways: users can directly input a SMILES string, draw the structure interactively using the embedded molecular editor, or upload a file containing multiple SMILES strings, with each line representing a unique structure. The tool includes a built-in structure viewer, allowing users to visualize the input molecules interactively and verify their accuracy before proceeding.

In addition to the substrate, users can specify one or more target proteins for the prediction workflow. DerivaPredict supports multi-protein input, making it suitable for projects involving polypharmacology or multitarget drug discovery. To simplify the process, users can input the gene names of the target proteins instead of their amino acid sequences. The software automatically retrieves the corresponding protein sequences from UniProt, ensuring accuracy and eliminating the need for manual sequence curation.

The parameter-setting panel within DerivaPredict offers users control over key aspects of the prediction process. Users can configure the method for derivative prediction, selecting from available chemical, biochemical, or metabolic transformation options. They can also specify the number of iterations for the derivative generation to explore the chemical space comprehensively. Additionally, users can choose the desired deep learning model for drug–target affinity (DTA) prediction, tailoring the computational pipeline to their specific research needs.

Once the parameters are set, users can run the workflow and save the results using the dedicated save button. The output includes comprehensive data, such as the structures of the generated derivatives, their predicted binding affinities, and detailed ADMET profiles. The results are stored in a user-friendly format, facilitating the downstream analysis and integration with experimental workflows.

### 2.3. Illustrative Examples

#### 2.3.1. Structural Diversity Evaluation

As a case study, we selected curcumin and paclitaxel as the parent compounds. Using chemical, biochemical, and metabolic transformation rules, a total of 1299 and 1497 unique derivatives were generated. For the chemical and biochemical transformations, the parameters were configured to perform two iterations. For the metabolic transformations, we selected environmental microbial transformation rules, with the parameters set to perform three iterations. Each iteration was designed to predict up to 30 derivatives. These derivatives spanned a broad chemical space, as visualized using Morgan fingerprints projected onto a U-Map, where the derivatives exhibited significant structural variation. Moreover, the chemical spaces covered by the different types of transformations exhibited distinct characteristics.

We compared the structural similarity between the derivatives and corresponding substrates and visualized the distribution trends using frequency distribution histograms. The chemical and biochemical transformations typically yielded derivatives with higher similarity to the substrates, as these processes primarily involved the addition or modification of functional groups. This is largely due to the prevalence of such reaction rules in transformation databases. In contrast, the metabolic transformations generated derivatives with greater structural diversity, often introducing more significant changes such as ring closures, chain elongations, or bond breakage. These distinctions underscore the unique capabilities of each transformation approach in exploring diverse regions of chemical space.

**Figure 3:**
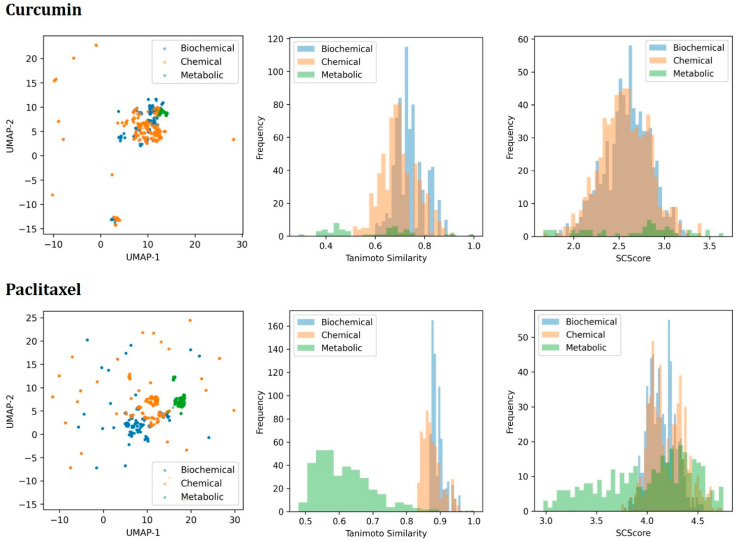
Visualization and analysis of generated derivatives. The U-Map projection illustrates the distribution of the derivatives in chemical space, highlighting the structural diversity (**left**). The Tanimoto similarity histograms depict the structural similarity between the derivatives and their parent compounds, showing the impact of the different transformation types (**middle**). The SCScore distributions compare the synthetic complexity of the derivatives, indicating variations based on the transformation methods and parent compound structures (**right**).

Considering synthetic complexity, the SCScore values for the derivatives of paclitaxel were generally higher than those for curcumin. This is likely due to the inherently more complex chemical structure of paclitaxel. For derivatives of the same substrate, the distributions of SCScore values for the chemical and biochemical transformations were relatively similar, while those for the metabolic transformations were more dispersed, reflecting greater structural variability (Figure 3).

#### 2.3.2. Pharmacological Active Prediction

Previous studies have demonstrated the potential inhibitory activity on the epidermal growth factor receptor (EGFR) of curcumin [33,34]. The pretrained CNN model was used to predict the affinity between the derivatives and their original target proteins [35]. As a demonstration, we utilized DerivaPredict’s functionality to explore the pharmacological potential of the generated derivatives by predicting their affinity for the EGFR using in silico docking methods. The goal was to showcase the software’s feature that predicts the binding potential of novel curcumin derivatives. While the algorithms and models used are derived from published works, benchmarking the accuracy of these predictions is beyond the scope of this study.

**Figure 4:**
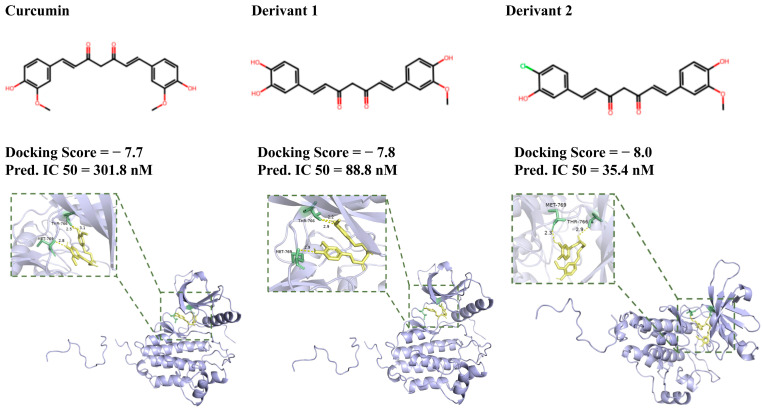
Structures, docking scores, and predicted IC50 of curcumin and its derivatives.

Among the derivatives, 737 were predicted to have an IC50 lower than that of curcumin. Two example chemical structures, with their predicted IC50 values, are shown in Figure 4, along with their predicted affinity for the EGFR, comparing them to curcumin. To validate these predictions, we employed AutoDock Vina to calculate the docking scores based on the three-dimensional structure of the EGFR (PDB ID: 1M17). We compared the binding modes of curcumin and the derivative with the highest predicted binding affinity in complex with the EGFR. Molecular docking simulations revealed that the derivative similarly binds the EGFR to curcumin. However, the derivative exhibited tighter binding compared to curcumin, likely due to additional favorable interactions such as stronger hydrogen bonding and hydrophobic interactions.

Notably, Derivative 1 (CHEMBL103410) is registered in the ChEMBL database, with the Max Phase listed as “Preclinical”. Although no experimental data specifically targeting the EGFR are available, prior in silico studies have validated that this compound has a higher binding affinity to the EGFR compared to curcumin. Additionally, the contact sites of the EGFR for this derivative align closely with our study, providing further evidence of its potential as an EGFR inhibitor [33,34].

Additionally, we evaluated the ADMET properties of curcumin and its derivatives using the integrated ADMET prediction function in DerivaPredict (Table 1). The logP values indicate that Derivate 1 is more lipophilic than curcumin, which could affect its bioavailability and membrane permeability. Both Derivate 1 and Derivate 2 show good ADMET profiles, with favorable QED values and bioavailability scores. Furthermore, the derivatives exhibit promising results in terms of the potential interactions with various cytochrome P450 enzymes (CYP1A2, CYP2C19, CYP3A4), suggesting a reasonable metabolic stability. Their predicted solubility and permeability (PAMPA and Caco2 scores) also indicate a good absorption potential. These findings suggest that some of the derivatives may exhibit enhanced stability and potency as EGFR inhibitors.

## 3. Materials and Methods

### 3.1. Extraction of Reaction Templates

DerivaPredict applies various types of in silico chemical, biochemical, or metabolic transformations to the substrate based on user-defined parameters. This process generates a range of potential derivative structures for the given substrate. The chemical transformations are based on the extraction reaction rule templates from 50,000 organic chemical reactions in the patent literature [36]. The biochemical transformations, in turn, are based on the reaction rule templates from 95,000 enzymatic reactions sourced from databases, including MetaCyc [37], KEGG [38], SEED [39], Rhea [40], and BiGG [41], as integrated by Zheng et al. [42]. Since the reactions in enzymatic reactions are not atom-mapped, RXNMapper [43], a neural network-based automated atom mapping model, was employed to generate an atom-mapped dataset. Reactions containing the wildcard of the ‘R’ token in their SMILES strings were excluded due to RXNMapper’s canonicalization process.

The metabolic transformations utilize the BioTransformer 3.0 module [44], which includes predictions for eight types of metabolic transformations: promiscuous enzymatic (EC) reactions, environmental microbial transformations, Phase I reactions (cytochrome P450), Phase II reactions, human gut microbial reactions, and various combinations of the above. These integrations enable the derivation of natural products through accessible chemical, enzymatic, microbial, and human metabolic transformations.

### 3.2. Generation of Potential Derivatives

To generate potential derivatives from natural products, DerivaPredict applies the reaction templates extracted in the previous step to the substrate using the RDKit library, a powerful cheminformatics toolkit. RDKit is employed to efficiently apply the applicable reaction rules, transforming the input natural product into a diverse set of potential derivatives based on the defined transformation templates.

The reaction templates, which include both chemical and biochemical transformations, are mapped to the substrate using RDKit’s reaction engine. This engine performs the transformations by breaking down the molecular structures into their component parts and applying the corresponding reaction rules. For example, if a chemical transformation rule suggests hydroxylation at a specific position, RDKit will modify the substrate molecule accordingly, creating new potential derivatives. The process ensures that only practicable reactions—those that can realistically occur given the structural constraints of the input molecule—are applied. DerivaPredict can also incorporate metabolic transformations by leveraging the BioTransformer 3.0 JDK module, which extends the tool’s capability to predict how the substrate might be modified through human metabolic processes or microbial reactions.

The in silico transformation can be iterated 1–3 times. Through this multi-step process, DerivaPredict generates a broad spectrum of potential derivatives, offering users a rich set of chemical entities for further evaluation in drug discovery workflows. As the number of iterations increases, the number of structures obtained increases exponentially, at the cost of consuming more time.

### 3.3. Prediction of Molecular Properties

DerivaPredict integrates a suite of advanced tools to predict and evaluate the molecular properties of the derivatives it generates, enabling comprehensive assessments of their potential as drug candidates.

To assess synthetic complexity, DerivaPredict employs the SCScore algorithm [45], which provides a quantitative measure of the ease or difficulty of synthesizing a compound. This metric helps users prioritize derivatives that are not only chemically novel but also synthetically feasible, an essential consideration in the early stages of drug development. To evaluate drug similarity, DerivaPredict uses the Quantitative Estimation of Drug-likeness (QED) metric. QED provides a composite score that reflects how closely a compound aligns with known drug-like properties, considering factors such as molecular weight, lipophilicity, and hydrogen bonding.

For a detailed evaluation of drug-like properties, DerivaPredict incorporates the ADMET-AI package [46]. This powerful tool predicts a comprehensive set of ADMET (absorption, distribution, metabolism, excretion, and toxicity) profiles, including six primary ADMET classes and 91 specific properties. These predictions encompass critical factors such as bioavailability, blood–brain barrier permeability, metabolic stability, and toxicity risks, providing a holistic view of each derivative’s pharmacokinetic and safety profiles.

### 3.4. Prediction of Binding Affinity with Specific Targets

Deep learning-based drug–target affinity (DTA) [47,48,49,50,51,52] and quantitative structure–activity relationship (QSAR) [53,54,55,56] prediction have been introduced by various studies. DerivaPredict incorporates several state-of-the-art deep learning models to predict the binding affinity of generated derivative structures against user-defined target proteins. These models leverage advanced architectures, such as convolutional neural networks (CNNs) and graph neural networks (GNNs), to provide accurate predictions of the half-maximal inhibitory concentration (IC50), a critical metric for evaluating the potency of potential drug candidates.

The predictive models used in DerivaPredict are pretrained using the DeepPurpose package with BindingDB datasets [35], a comprehensive resource containing experimentally validated binding affinities for a wide range of small molecules and protein targets. This pretraining ensures that the models are well-optimized for analyzing diverse chemical structures and biological targets, enabling reliable predictions even for novel derivatives. This functionality not only accelerates the screening process but also empowers users to make data-driven decisions in prioritizing compounds for further investigation.

### 3.5. User-Defined Parameters and Initial Settings

DerivaPredict provides a variety of user-configurable parameters to customize the molecular generation and evaluation processes. Users can define the number of transformation steps, with a default setting of two, which is also applied in the case study of this work, and an adjustable range between one and three. They can also select reaction types, including chemical, biochemical, and metabolic transformations. For the binding affinity assessments, DerivaPredict enables users to input target proteins using a UniProt ID or gene name, allowing for precise molecular designs.

### 3.6. Molecular Docking

The X-ray crystallography-based three-dimensional structure of the EGFR was obtained from the RCSB Protein Data Bank (PDB ID: 1M17; accessed on 26 September 2024) and used as a docking template throughout the calculations. The two-dimensional structures of curcumin and its derivatives were energy-minimized using the MMFF method and subsequently converted to 3D structures using the RDKit package (v2022.09.3) for compatibility with the docking operations. Molecular docking was performed using AutoDock Tools (1.5.7), with the docking center coordinates set at (23.568, 9.824, 59.369). The docking procedure was carried out independently three times, generating 30 distinct conformations. Finally, PyMOL (2.6.0) was used to visualize and further illustrate the binding modes obtained from the docking analysis.

## 4. Conclusions

In summary, DerivaPredict serves as a rational design engine that systematically generates candidate molecular structures for drug screening while prioritizing biologically relevant derivatives of natural products. Its core innovation lies in bridging two critical phases of drug discovery: (1) the rational generation of chemically feasible derivatives through biotransformation-aware algorithms and (2) the prioritization of screening candidates via integrated target affinity predictions and automated ADMET evaluation workflows.

By focusing on rule-compliant structural diversification, the tool addresses the challenge of expanding natural products’ chemical space beyond existing database limitations, providing medicinal chemists with pre-filtered compound libraries that balance novelty and drug-likeness. Crucially, DerivaPredict operates as a hypothesis generator—its machine learning models identify high-potential derivatives for experimental validation rather than claiming to replace wet-lab studies. By offering a user-friendly interface and automated workflows, DerivaPredict empowers researchers to generate and evaluate derivative structures without the need for extensive computational expertise, accelerating the discovery of bioactive compounds in natural products research.

## Figures and Tables

**Table 1:** Predicted ADMET properties of curcumin and its derivatives.

	Curcumin	Derivate 1	Derivate 2
MW	368.385	372.804	354.358
SCScore	1.804	2.109	2.212
logP	3.370	4.015	3.067
nHBA	6	5	6
nHBD	2	2	3
QED	0.548	0.566	0.401
TPSA	93.060	83.830	104.060
BBB	0.344	0.255	0.286
BS	0.463	0.578	0.414
CYP1A2 inhibition	0.439	0.665	0.420
CYP2C19 inhibition	0.582	0.703	0.506
CYP3A4 inhibition	0.751	0.771	0.697
PAMPA	0.739	0.734	0.663
Caco2	−5.171	−5.084	−5.283

MW: molecular weight, SCScore: synthetic complexity score, logP: log of octanol/water partition coefficient, nHBA: number of hydrogen bond acceptor(s), nHBD: number of hydrogen bond donor(s), TPSA: total polar surface area, BBB: blood–brain barrier permeant, BS: bioavailability score, CYP2C19/CYP1A2/CYP3A4: inhibition potential for corresponding enzyme, PAMPA: parallel artificial membrane permeability assay, Caco2: Caco2 cell permeability.

## Data Availability

DerivaPredict is written by Python 3.10 and PyQt5, which is a stand-alone software that can be run on cross-platforms. The manual and tutorial videos as well as example data can be found in our GitHub repository (https://github.com/hcji/DerivaPredict, accessed on 4 January 2025).
